# Evolutionary game analysis of rural public–private partnership older adult care project in the context of population aging in China

**DOI:** 10.3389/fpubh.2023.1110082

**Published:** 2023-08-23

**Authors:** Jianru Fu, Chao Huang, Shicheng Li, Yihan Xia

**Affiliations:** ^1^Management Science and Engineering Research Center, Jiangxi Normal University, Nanchang, Jiangxi, China; ^2^College of Finance and Economics, Jiangxi Normal University, Nanchang, Jiangxi, China

**Keywords:** population aging, rural older adult care, public-private older adult care projects, action strategies, evolutionary game analysis

## Abstract

**Introduction:**

Public–private partnership (PPP) older adult care project is an effective means to solve the dilemma of the aging population in China's rural areas, but there are some problems in the operation process, such as a low participation rate and poor service quality, resulting in the needs of rural older adult groups not being met.

**Methods:**

To alleviate the pressure of the aging population in rural areas, this study establishes an evolutionary game model for the PPP older adult care project, then defines the interests of local government, the private sector, and rural older adult residents, based on which it discusses the strategic choices of the three parties in the evolutionary process, and finally analyzes the influencing factors of the strategic choices of the game parties through simulation.

**Results:**

The results suggest that whether the private sector chooses to actively participate in the project will be influenced by the willingness of local government and rural older adult residents to participate in the project. Local government could play the role of supervisor through reward and punishment mechanisms. Whether older rural residents choose to participate in the project depends on the number of benefits they would receive.

**Discussion:**

Based on these findings, local governments should clarify the responsibilities of relevant stakeholders, adopt a regulatory strategy combining subsidies and penalties, improve the participation efficiency of rural older adult residents, promote the effective operation of PPP older adult care projects, and improve the quality of rural older adult care services in the new era.

## Introduction

Population aging is a complex issue that the world must deal with in the twenty first century. According to the United Nations, the global population over the age of 65 was 702.9 million in 2019 and is expected to rise to 1.55 billion by 2050 (1). Population aging will also be a fundamental national situation in China for a long time in future, and actively providing care services for the older adult has a significant impact on the overall situation of national development and the wellbeing of hundreds of millions of people (2). With the urbanization of the rural young adult workforce and the miniaturization of family structures, the traditional function of family care has been weakened (3). Rural institutions for the older adult are still insufficient, the new rural older adult care system has not yet been established, and there are still many problems in the economic and spiritual lives of the rural older adult (4). Under these conditions, it is of great practical significance to study how to effectively provide services for the rural older adult and establish a sound protection system for them.

The key issue in establishing a proper rural older adult security system lies in the choice of the rural older adult operation model (5). In current practice, there are three main ways to provide older adult care in China's rural areas: home-based older adult care, community-based older adult care, and institution-based older adult care. With the accelerating process of urbanization and the continuous outflow of the rural population, traditional home-based and community-based older adult care can no longer meet the needs of the older adult population, and the private older adult care service model is receiving more attention (6). In 2017, China started to try to develop public–private older adult care projects to improve rural older adult care services (7). The rapid growth of the rural older adult population not only brings opportunities for the development of the public–private partnership (PPP) older adult care industry but also raises great challenges for policymakers in the provision of older adult care services (8). For example, the problem of information asymmetry between the private sector and government departments can easily lead to problems such as single content and unprofessional services for the older adult, which in turn increase the risk of older adult care for the rural older adult population. Therefore, government regulation policies are needed to ensure high-quality older adult care services in the PPP older adult care project (9).

Government regulation measures for traditional rural older adult care projects have relied mainly on government subsidies. For example, China's Old Age Pension Fund project and Japan's Rural In-Home Pension project are both initiated and managed by local governments, but both have encountered economic challenges in the regulatory process (10). PPP older adult care projects can reduce the government's financial pressure compared to traditional rural older adult care projects, but the private sector is also prone to speculative behavior. So, some scholars have pointed out that the government needs to adopt a combination of incentives and penalties for PPP projects, rewarding the private sector for providing quality services and punishing the private sector for opportunistic behavior (11). Sabry pointed out that how and when to implement the monitoring strategy or subsidy strategy has a decisive impact on the success or failure of PPP cooperation (12). As a new form of cooperation between the government and private sector, PPP projects can provide the public with quality and efficient public goods or services (13). The application of a PPP project in the rural older adult care sector is an innovative application in the context of China's aging population, but it is still in the exploratory stage (14). Due to the strong public welfare attributes of PPP older adult care projects, the government is prone to over-manage or give up the supervision of the projects, and the private sector is prone to focus on the short-term benefits and neglect the sustainable development of the projects (15). Only by balancing the interests of multiple stakeholders and seeking the greatest possible consensus can PPP older adult care projects be guaranteed to operate in a stable way (16). However, few studies concerning older adult care services have been conducted on how to subsidize service providers and the public to achieve project success (17). So, how to coordinate the interests of all parties involved in public–private older adult care projects through government regulation strategies and how to promote the development of rural public–private older adult care projects of higher quality in the context of population aging are all issues that need to be studied.

This study analyzes the decision-making processes of the government and the private sector in the provision of elder care services in a long-term and dynamic environment. A multi-agent evolutionary game model is established with the assumption that participants display bounded rationality (18). The evolutionary equilibrium of the complex system is obtained through theoretical analysis. The effects of different parameters on the evolution of strategies among participants under different scenarios are investigated. This article tries to answer the following research question: In the process of long-term dynamic structuring of rural public–private older adult care service provision, the government can adjust the level of regulation by increasing the incentives for positive cooperation, subsidies for active participation, and penalties for negative participation in the private sector. So, under different conditions, what regulatory strategies will the government adopt? Under what conditions is the private sector willing to provide quality services? What policies and measures should the government adopt to encourage the private sector to provide high-quality services and to ensure the active participation of rural residents in rural PPP older adult care projects?

Compared with the existing related research literature, we can see that the innovations in this study are as follows. First, it expands the traditional two-subject research paradigm of government and private sector and takes rural residents as a whole subject, which enriches the existing research topics. Second, from the perspective of an evolutionary game, it uses numerical simulation to explore the evolutionary process of governmental regulatory behavior more deeply, providing theoretical support for governmental regulation of rural PPP older adult care projects. Third, the introduction of the tripartite initial willingness of the system and the consideration of the impact of the initial state of the system are an expansion of the existing rural PPP older adult care model and enrich its practical significance.

The remainder of this study is structured as follows: in the “Literature review” section, the relevant literature on the application of rural older adult care services and the application of evolutionary game theory is reviewed. In the “Evolutionary game model” section, hypotheses and mathematical models are presented to develop behavioral strategies for the tripartite participants. In the “Numerical simulation results” section, the impacts and results of the initial willingness of the tripartite participants and the different strategies adopted by the participants in the evolutionary process are presented through numerical simulation analysis. Finally, brief conclusions and suggestions are given in the “Conclusions and policy suggestions” section.

## Literature review

This research is related to two main streams of literature. First, from the perspective of the research question, it was linked to rural older adult care services and their provision. Second, from a modeling perspective, it is relevant to the application of evolutionary games.

### Rural older adult care services and their provision

Rural older adult care service is one of the hot topics in current academic research. In the current development of the older adult care business, there are outstanding problems in some areas, such as the lack of policy implementation and insufficient capital investment (19). Su et al. (20) conducted a study on the issue of “who is going to support the older adult” in China (20). He found that home-based older adult care is weakening, while the development of government older adult care services is still in its infancy and quite weak. At present, there are still some dilemmas in governmental older adult care, which cannot replace family older adult care for time being (21). It is believed that older adult care services provided by the government can greatly enhance the sense of social participation of the older adult. There are three operational forms of government-provided older adult care services in rural areas: embedded older adult care services, mutual aid older adult care services, and PPP older adult care services. Among these three kinds of older adult care services, embedded older adult care services bring greater economic pressure on the government, while mutual aid older adult care services make it difficult to meet the multiple needs of rural older adult residents. PPP older adult care services can reduce the government's economic pressure and, on the contrary, can provide a wide variety of older adult care services, so this kind of older adult care service is a more desirable form. Moreover, Lee and Tan (22) further pointed out that the form of PPP in the older adult care field can promote the active participation of the older adult (22). In such projects, older people can actively participate in the process of older adult care services according to their conditions and needs, which greatly increases their sense of social participation.

Researchers have also studied factors that affect the provision of PPP older adult care services. Among the many older adult care services provided by the Government, the PPP older adult care project has more development potential given the current situation and prospects of rural older adult care development. PPP older adult care projects can integrate older adult care resources in the surrounding area and provide specialized and personalized services for the older adult in the neighborhood. Chapman et al. (23) pointed out that market-based rural aging projects can fully integrate existing health and aging resources (23). Through an analysis of Japan's “Time Bank” care project, Kim found that the private sector's support for the older adult plays an important role in compensating for the current shortage of older adult care workers and the weakening of traditional family care functions (24). Similarly, Morgan also said that rural older adult services with private sector involvement could provide more human resources for the older adult (25). However, there are several unresolved problems in the operation of public–private older adult care projects. One is that service providers are prone to moral hazard issues that lower the standards of the services they provide. Xia (26) found that the participation rate of the older adult in PPP older adult care services is low, and satisfaction with service accessibility remains to be improved (26). Sudo et al. (27) argued that older adult care organizations are in a powerful position to provide services and may engage in speculative behavior, making what is offered inconsistent with the needs of residents (27). The other is that it is difficult for the government to regulate older adult care services. Musenerol et al. (28) found that appropriate government regulation can effectively promote the healthy development of infrastructure PPP projects (28). Braun et al. (29) also pointed out that market-based policies have difficulty playing a decisive role in the resource allocation of older adult care services, which was proved by the evidence of the low motivation of participants (29). In addition, in the PPP older adult care project, public participation can not only promote government efficiency but also affect the effective operation of the project. To address this issue, Zhang (30) suggested that the specific implementation of the older adult care service market needs to further clarify the interest coordination mechanism, establish a compensation payment mechanism, and ensure sustainable collaborative participation of multiple parties (30).

In the PPP older adult care project, previous studies have focused on how the government develops and regulates the provision of older adult care services, with less discussion of other actors. The government plays the role of supervisor and is responsible for regulating and managing the operation of the PPP older adult care project. Yang et al. (31) pointed out that the PPP older adult care project faces a series of problems (31), such as organizational irregularities and communication difficulties, which need to be adjusted and regulated. Huang and Zhang (32) emphasized that policy guarantee is an important factor for the effective operation of older adult care projects (32). Speck (33) also pointed out that the development of older adult care projects should be based on sound organizational norms and should be adapted to local conditions (33). Therefore, Shi and Hu (34) suggested that the current older adult care service needs further adjustment and standardization by the community in terms of top-level design, financial guarantee, organizational management, and local construction (34).

Overall, previous quantitative and qualitative studies analyzed the sustainable provision of older adult care services from the perspectives of policy optimization, price, and quality competition. There are few studies concerning the strategic interaction between decision-makers associated with service provision.

### Evolutionary game theory and its application

Game theoretic approaches can provide a quantitative decision framework for modeling, analyzing, and predicting the behaviors of the players in the game (35). For instance, Debreu included consumers as decision-makers in the economic system to study the equilibrium of the game model (36). Game theory has been used to study decision-making among multiple actors, such as in the recycling of construction waste. Li et al. (37) found that government regulation plays an important role in PPP projects through a study of the green innovation behaviors of construction companies (37). Liu et al. (38) elucidated the relationship between government regulation and firm participation through a game study of manufacturers “Green-washing” behaviors (38). Zou et al. (39) studied the evolutionary game of government participation in low-carbon technology innovation to provide a reference for further improving the theory of green development (39). Dai et al. (40) used game theory to study the vaccine supply chain from a long-term, dynamic perspective and found that the effective participation of residents is important (40).

To sum up, game theory has been used in many studies to analyze decision-making among multiple subjects, but relatively few studies have applied game theory to rural older adult care projects. This study uses evolutionary game theory to construct a tripartite game model among local government, private sector, and rural residents in the rural PPP older adult care project, then introduces the initial willingness of local government, private sector, and rural residents into the model to explore the influence on the system evolution by the initial state of the system. Then software was used to simulate the evolution of local government, private sector, and rural residents to explore the application of the PPP model in rural older adult care services, from which suggestions were made to promote the development of the PPP model for rural older adult care services.

## Evolutionary game model

### Descriptions and hypotheses

The PPP older adult care project involves key subjects such as local governments, the private sector, and rural residents. To study the evolution of cooperative behavior strategies of related subjects in the PPP model, the specific hypotheses are as follows, and the relevant game variables are shown in Table 1.

**Table 1:** Variables and definitions.

**Variables**	**Mean of variables**	**Variables**	**Mean of variables**
*R*_*g*_	The basic benefits obtained by the government	*R*_*f*_	The income of residents when they chose not to participate in the project
*C*_*g*_	The basic expenditures of the government	*R*_*j*_	The income of residents when they chose to participate in the project
*C*_*r*_	The supervision cost of the government	*L*_*s*_	The cost of rural residents when they chose to participate in the project
*R*_*ge*_	The additional income of the government by active regulation	*C*_*m*_	The cost to residents of supervising the behavior of the private sector
*L*_*g*_	The loss to local governments caused by the private sector's speculation	*L*_*s*_	The losses of the private sector when speculation is discovered by residents
*R*_*s*_	The basic income of the private sector from the project	α*S*	The local government subsidies for active cooperation of the private sector
*C*_*s*_	The cost invested in the project of the private sector	β*R*	The reward for the private sector according to the completed result
*R*_*se*_	The additional income of the private sector by negative cooperative	γ*P*	The punishment for the private sector's speculative behaviors
*C*_*P*_	The cost invested in the project of the private sector by negative cooperative	δ*T*	The rewards for residents to participate in the project

***Hypothesis 1:*** Local government, private sector, and rural residents constitute a complete system. Assuming that the three participants are limited rational individuals and that there is an incomplete symmetry of information between the parties, other subjects in the PPP project that may have an impact on the game system are not considered in the game process.

***Hypothesis 2:*** The local government, as the leader of the rural PPP older adult care project, bears the responsibility of guiding the private sector to actively cooperate and supervise their speculation. Their strategic choices are positive regulation or negative regulation. As the service providers for the PPP older adult care project, the private sector's strategy choice is active cooperation or passive cooperation. As service recipients of the PPP older adult care project, rural residents can actively participate in the construction of rural PPP older adult care projects and can also “hitchhike” to enjoy the benefits of improving older adult care facilities. Their strategic choice is whether to participate in a project or not. The probability that local governments choose positive regulation is, the probability that the private sector choose active cooperation is *y*, and the probability that residents choose to participate is *z*.

***Hypothesis 3:*** The basic benefits obtained by the local government in the project are defined as *R*_*g*_, and the cost of adopting a regulation strategy is defined as *C*_*r*_. *C*_*g*_ denotes the basic expenditure of the local government in the PPP older adult care project. *R*_*ge*_ represents the additional income from the PPP older adult care project under the positive regulation of the government. *L*_*g*_ denotes the loss to local governments caused by the private sector when the private sector adopts a negative cooperation strategy.

***Hypothesis 4:*** The basic income of the private sector from the project, which we defined as *R*_*s*_. *C*_*s*_ represents the cost of the private sector that invested in the project. *R*_*se*_ represents the additional income gained by the private sector through negative cooperative behaviors. And we assume the cost of speculation as *C*_*p*_. Moreover, α*S*denotes the subsidy provided by the local government for the private sector in project implementation, α represents the subsidy intensity, and *S* assumes the upper limit of subsidy. When the private sector takes the strategy of actively cooperating, *R*_*f*_ was defined as the reward for the private sector according to the project completion result, β represents the reward intensity, and *R* is classified as the upper limit of the reward. When the government takes the strategy of positive encouragement and regulation, it will supervise and penalize the private sector that violates regulations or other speculative practices, which we defined as γ*P*. γ represents the punishment intensity, and *P* is classified as the upper limit of the punishment. The loss caused by residents' supervision and reporting of private sector speculation was denoted as *L*_*s*_.

***Hypothesis 5:*** When rural residents take the strategy of not participating in the construction of the rural PPP older adult care project, they do not pay the cost of participation but benefit from the project's construction through “hitchhiking,” which was defined as *R*_*f*_. However, when rural residents choose a strategy to participate in the project, their incomes are classified as *R*_*j*_(*R*_*j*_>*R*_*f*_). In addition,*C*_*f*_ was defined as the cost of participation of rural residents in the project. Residents would supervise the private sector's speculation behavior after they joined the project, and the cost of supervision was denoted as *C*_*m*_. When the government adopts an active regulation strategy, residents participate in the project and supervise the services provided by the private sector. The local government will give rewards to the residents, which we defined as δ*T*. δ represents the incentive intensity and *T* represents the upper limit of rewards.

### Game payment matrix and strategy solution

Based on the above analysis, a tripartite evolutionary game payment matrix is constructed for local government, private sector, and residents, and the game payment matrix is shown in Table 2 when the local government adopts a positive regulatory strategy or Table 3 when the local government adopts a negative regulatory strategy.

**Table 2:** Payment matrix of a three-party evolutionary game with positive government regulation.

		**Residents**	
		**Participate in projects (*z*)**	**Not participate in projects (1−*z*)**
Private sector	Active cooperation (*y*)	*R*_*g*_+*R*_*ge*_−*C*_*r*_−*C*_*g*_−α*S*−β*R*−δ*T* *R*_*s*_−*C*_*s*_+α*S*+β*R* *R*_*j*_−*C*_*f*_−*C*_*m*_+δ*T*	*R*_*g*_+*R*_*ge*_−*C*_*r*_−*C*_*g*_−α*S*−β*R* *R*_*s*_−*C*_*s*_+α*S*+β*R* *R*_*f*_
	Passive cooperation (1−*y*)	*R*_*g*_−*C*_*r*_−*C*_*g*_−*L*_*g*_+γ*P*−δ*T* *R*_*s*_+*R*_*se*_−*C*_*p*_−*C*_*s*_−γ*P*−*L*_*s*_ *R*_*j*_−*C*_*f*_−*C*_*m*_+δ*T*	*R*_*g*_−*C*_*r*_−*C*_*g*_−*L*_*g*_+γ*P* *R*_*s*_+*R*_*se*_−*C*_*p*_−*C*_*s*_−γ*P* *R*_*j*_

The formulas from top to bottom represent payments by the government, private sector, and rural older adult residents, respectively.

**Table 3:** Payment matrix of a three-party evolutionary game with negative government regulation.

		**Residents**	
		**Participate in projects (*z*)**	**Not participate in projects (1−*z*)**
Private sector	Active cooperation (*y*)	*R*_*g*_−*C*_*g*_−α*SR*_*s*_−*C*_*s*_+α*SR*_*j*_−*C*_*f*_−*C*_*m*_	*R*_*g*_−*C*_*g*_−α*SR*_*s*_−*C*_*s*_+α*SR*_*f*_
	Passive cooperation (1−*y*)	*R*_*g*_−*C*_*g*_−*L*_*g*_*R*_*s*_+*R*_*se*_−*C*_*p*_−*C*_*s*_−*L*_*s*_*R*_*j*_−*C*_*f*_−*C*_*m*_	*R*_*g*_−*C*_*g*_−*L*_*g*_*R*_*s*_+*R*_*se*_−*C*_*p*_−*C*_*s*_*R*_*j*_

The formulas from top to bottom represent payments by the government, private sector, and rural older adult residents, respectively.

Combined with the above payment matrix, the expected payoffs of the game subjects under different behavioral strategies can be derived as follows:

The expected return and average expected return of the government's choices of positive regulation or negative regulation can be expressed as follows:


(1) $$\begin{array}{l} \begin{array}{c} E_{gp}=yz\left(R_{g}+R_{ge}-C_{r}-C_{g}-\alpha S-\beta R-\delta T\right)\\ \;+y\left(1-z\right)\left(R_{g}+R_{ge}-C_{r}-C_{g}-\alpha S-\beta R\right)\\ \;+\left(1-y\right)z\left(R_{g}-C_{r}-C_{g}-L_{g}\gamma P-\delta T\right)\\ \;+\left(1-y\right)\left(1-z\right)\left(R_{g}-C_{r}-C_{g}-L_{g}+\gamma P\right) \end{array} \end{array}$$

(2) $$\begin{array}{l} \begin{array}{c} E_{gn}=yz\left(R_{g}-C_{g}-\alpha S\right)\\ \;+y\left(1-z\right)\left(R_{g}-C_{g}-\alpha S\right)\\ \;+\left(1-y\right)z\left(R_{g}-C_{g}-L_{g}\right)\\ \;+\left(1-\text{y}\right)\left(1-z\right)\left(R_{g}-C_{g}-L_{g}\right) \end{array} \end{array}$$

(3) $$\begin{array}{l} E_{g}=xE_{gp}+\left(1-x\right)E_{gn} \end{array}$$

The replication dynamic equation of local government can be derived as:


(4) $$\begin{array}{l} G_{\left(x\right)}=\frac{dx}{dt}=x\left(E_{gp}-E_{g}\right)=x\left(1-x\right)[y\left(R_{ge}-\beta R\gamma P\right)\\ -C_{r}+\gamma P-z\delta T] \end{array}$$

The expected return and average expected return of the private sector's choices of active cooperation or passive cooperation can be expressed as follows:


(5) $$\begin{array}{l} \begin{array}{c} E_{sa}=xz\left(R_{s}-C_{s}+\alpha S+\beta R\right)\\ \;+x\left(1-z\right)\left(R_{s}-C_{s}+\alpha S+\beta R\right)\\ \;+\left(1-x\right)z\left(R_{s}-C_{s}+\alpha S\right)\\ \;+\left(1-x\right)\left(1-z\right)\left(R_{s}-C_{s}+aS\right) \end{array} \end{array}$$

(6) $$\begin{array}{l} E_{sp}=xz\left(R_{s}+R_{se}-C_{p}-C_{s}-\gamma P-L_{s}\right)\\ \;+x\left(1-z\right)\left(R_{s}+R_{se}-C_{p}-C_{s}-\gamma P\right)\\ \;+\left(1-x\right)z\left(R_{s}+R_{se}-C_{p}-C_{s}-L_{s}\right)\\ \;+\left(1-x\right)\left(1-z\right)\left(R_{s}+R_{se}-C_{p}-C_{s}\right) \end{array}$$

(7) $$\begin{array}{l} E_{s}=yE_{sa}+\left(1-y\right)E_{sp} \end{array}$$

The replication dynamic equation of the private sector can be derived as:


(8) $$\begin{array}{l} G\left(y\right)=\frac{dy}{dt}=y\left(E_{sa}-E_{s}\right)\\ \;=y\left(1-y\right)\left[x\left(\beta R+\gamma p\right)+zL_{s}+\alpha S+C_{p}-R_{se}\right] \end{array}$$

The expected return and average expected return of residents who choose to participate in the projects or not participate in the projects can be expressed as follows:


(9) $$\begin{array}{l} \begin{array}{c} E_{fj}=xy\left(R_{j}-C_{f}-C_{m}+\delta T\right)\\ \;+x\left(1-y\right)\left(R_{j}-C_{f}-C_{m}+\delta T\right)\\ \;+\left(1-x\right)y\left(R_{j}-C_{f}-C_{m}\right)\\ \;+\left(1-x\right)\left(1-y\right)\left(R_{j}-C_{f}-C_{m}\right) \end{array} \end{array}$$

(10) $$\begin{array}{l} E_{fr}=xyR_{f}+x\left(1-y\right)R_{f}+\left(1-x\right)yR_{f}\\ \;+\left(1-x\right)\left(1-y\right)R_{f} \end{array}$$

(11) $$\begin{array}{l} E_{f}=zE_{fj}+\left(1-z\right)E_{fr} \end{array}$$

The replication dynamic equation of residents can be derived as:


(12) $$\begin{array}{l} G\left(\text{z}\right)=\frac{dz}{dt}=z\left(E_{fj}-E_{f}\right)=z\left(1-z\right)\\ \left(R_{j}-R_{f}-C_{f}-C_{m}+x\delta T\right) \end{array}$$

### Stability analysis of tripartite game

According to formula (4), it can be derived that *y*(*R*_*ge*_−β*R*−γ*P*)−*C*_*r*_+γ*P*−*zδT* = 0, *G*′(*x*)equals 0, means the government's strategy is stable no matter which one it adopts. In the case of*y*(*R*_*ge*_−β*R*−γ*P*)−*C*_*r*_+γ*P*−*zδT*>0, we can show that *G*′(*x*) < 0 when *x* = 1, which represents that *x* = 1 is the evolutionary game equilibrium point, the government will adopt a positive regulation strategy. In the case of *y*(*R*_*ge*_−β*R*−γ*P*)−*C*_*r*_+γ*P*−*zδT* < 0, we can show that *G*′(*x*) < 0 when *x* = 0, which represents that *x* = 0 is the evolutionary game equilibrium point. According to formula (8), it can be derived that when *x*(β*R*+γ*p*)+*zL*_*s*_+α*S*+*C*_*p*_−*R*_*se*_ = 0, equals 0, which means that the private sector's strategy is stable no matter which one it adopts. Similarly, in this case of *x*(β*R*+γ*p*)+*zL*_*s*_+α*S*+*C*_*p*_−*R*_*se*_>0, we also show that *G*′(*y*) < 0 when *y* = 1 is the evolutionary game equilibrium point, the private sector will adopt an active cooperative strategy. In the case of *y*(*R*_*ge*_−β*R*−γ*P*)−*C*_*r*_+γ*P*−*zδT* < 0, we can show that *G*′(*x*) < 0 when *x* = 0, which represents that *x* = 0 is the evolutionary game equilibrium point. According to formula (12), it can be derived that when, *G*′(*z*) equal to 0, that means the residents' strategy is stable no matter which one it adopts. In the same way, we can show that *G*′(*z*) < 0 when *z* = 1, which means that rural residents choose to participate in the PPP older adult care project is the optimal strategy.

According to the analysis of formula (4), formula (8), and formula (12), we can obtain the local equilibrium points of the system. And the Jacobian matrix of the three-dimensional dynamics system is:


$$\begin{array}{l} J=\left[\begin{array}{c} J_{11}J_{12}J_{13}\\ J_{21}J_{22}J_{23}\\ J_{31}J_{32}J_{33} \end{array}\right] \end{array}$$

where *J*_11_ = (1 − 2*x*)(*y*(*R*_*ge*_−β*R*−γ*P*)−*C*_*r*_+γ*P*−*zδT*),


$$\begin{array}{l} J_{12}=x\left(1-x\right)\left(R_{ge}-\beta R-\gamma P\right),J_{13}=-x\left(1-x\right)\delta T,\\ J_{21}=y\left(1-y\right)\left(\beta R+\gamma P\right),\\ J_{22}=\left(1-2y\right)\left(x\left(\beta R+\gamma p\right)+zL_{s}+\alpha S+C_{p}-R_{se}\right),\\ J_{23}=y\left(1-y\right)L_{s},\\ J_{31}=z\left(1-z\right)\delta T,J_{32}=0,\\ J_{33}=\left(1-2z\right)\left(R_{j}-R_{f}-C_{f}-C_{m}+x\delta T\right). \end{array}$$

Based on the Jacobian matrix, we can conclude eight fixed points as equilibrium, namely,*E*_2_(0, 1, 0), *E*_3_(0, 0, 1), *E*_4_(1, 0, 0), *E*_5_(1, 1, 0), *E*_6_(1, 0, 1), *E*_7_(0, 1, 1), and *E*_8_(1, 1, 1), and one mixed equilibrium $E_{9}\left(x^{*},y^{*},z^{*}\right)$. According to Lyapunov stability (41), the equilibrium point is the evolutionary stable point of the system if the eigenvalues of the Jacobian matrix are non-positive numbers. For instance, the Jacobian matrix can be derived as follows in the case of *E*_1_(0, 0, 0).


$$J_{1}=\left[\begin{array}{c} -C_{r}+\gamma p00\\ 0\alpha S+C_{p}-R_{se}0\\ 00R_{j}-R_{f}-C_{f}-C_{m} \end{array}\right]$$

In the same way, the eigenvalues of equilibrium points are derived in Table 4. It is noted that mixed strategy equilibrium must not be an evolutionary stable equilibrium in the model of asymmetric games. Therefore, it is sufficient to discuss the asymptotic stability of pure strategy equilibrium.

**Table 4:** Equilibrium points and characteristic values.

**Equilibrium points**	**λ_1_**	**λ_2_**	**λ_3_**
*E*_1_(0, 0, 0)	−*C*_*r*_+γ*P*	α*S*+*C*_*p*_−*R*_*se*_	*R*_*j*_−*R*_*f*_−*C*_*f*_−*C*_*m*_
*E*_2_(0, 1, 0)	*R*_*ge*_−β*R*−*C*_*r*_	−α*S*−*C*_*p*_+*R*_*se*_	*R*_*j*_−*R*_*f*_−*C*_*f*_−*C*_*m*_
*E*_3_(0, 0, 1)	−*C*_*r*_+γ*P*−δ*T*	*L*_*s*_+α*S*+*C*_*p*_−*R*_*se*_	−*R*_*j*_+*R*_*f*_+*C*_*f*_+*C*_*m*_
*E*_4_(1, 0, 0)	*C*_*r*_−γ*P*	β*R*+γ*P*+α*S*+*C*_*p*_−*R*_*se*_	*R*_*j*_−*R*_*f*_−*C*_*f*_−*C*_*m*_+δ*T*
*E*_5_(1, 1, 0)	−*R*_*ge*_+β*R*+*C*_*r*_	−β*R*−γ*P*−α*S*−*C*_*p*_+*R*_*se*_	*R*_*j*_−*R*_*f*_−*C*_*f*_−*C*_*m*_+δ*T*
*E*_6_(1, 0, 1)	*C*_*r*_−γ*P*+δ*T*	β*R*+γ*P*+*L*_*s*_+α*S*+*C*_*p*_−*R*_*se*_	−*R*_*j*_+*R*_*f*_+*C*_*f*_+*C*_*m*_−δ*T*
*E*_7_(0, 1, 1)	*R*_*ge*_−β*R*−*C*_*r*_−δ*T*	−*L*_*s*_−α*S*−*C*_*p*_+*R*_*se*_	−*R*_*j*_+*R*_*f*_+*C*_*f*_+*C*_*m*_
*E*_8_(1, 1, 1)	−*R*_*ge*_+β*R*+δ*T*+*C*_*r*_	−β*R*−γ*P*−*L*_*s*_−α*S*−*C*_*p*_+*R*_*se*_	−*R*_*j*_+*R*_*f*_+*C*_*f*_+*C*_*m*_−δ*T*

Based on the equilibrium points and characteristic values, stable points and evolutionary strategies can be analyzed. To facilitate the analysis of eigenvalue symbols corresponding to different equilibrium points, it is assumed that *R*_*ge*_−β*R*−δ*T*−*C*_*r*_>0, *R*_*j*_−*R*_*f*_−*C*_*f*_−*C*_*m*_+δ*T*, α*S*+*C*_*p*_−*R*_*se*_>0. That is, the benefits brought by local governments, private sector, and rural older adult groups when they choose active participation strategies are greater than the benefits when they choose non-participation strategies. Due to the large number and complexity of parameters in the model, the evolutionary game stabilization strategies are discussed in the following three scenarios.

**Scenario 1**. If −*C*_*r*_+γ*P*−δ*T*>0, the equilibrium point *E*_8_(1, 1, 1) is an ESS, which means that governments positively supervise the quality of older adult care services, whereas the quality of services provided by the private sector is excellent, the rural older adult groups actively participate in PPP older adult care projects.

**Scenario 2**. If −*C*_*r*_+γ*P*>0 and −*C*_*r*_+γ*P*−δ*T* < 0, the equilibrium point *E*_8_(1, 1, 1) is an ESS, which means that governments positively supervise the quality of older adult care services, whereas the quality of services provided by the private sector is excellent, the rural older adult groups actively participate in PPP older adult care projects.

**Scenario 3**. If −*C*_*r*_+γ*P* < 0, the equilibrium point *E*_8_(1, 1, 1) is an ESS, which means that governments positively supervise the quality of older adult care services, whereas the quality of services provided by the private sector is excellent, the rural older adult groups actively participate in PPP older adult care projects.

## Numerical simulation results

It is impossible to intuitively reflect how the parameters in the system affect the evolution and stability of the system by analyzing and deducing the model only from the theoretical level. Therefore, this study uses MATLAB 2017 to simulate the evolution track of the participants' strategies, portray the initial willingness of the three players in the game, and examine the evolutionary impact of government regulation policies on the behavior strategies of the participants in the PPP older adult care project.

Referring to the setting of the initial value of the system by scholars such as Liu et al. (42) in the literature, in this study, the willingness of the private sector to participate is replaced with the amount of capital invested by the private sector, and the willingness of residents to participate as well as the strength of government regulation can be derived. The willingness of local governments to regulate, the private sector to cooperate, and rural residents to participate is set into three levels: 0.8 represents a high level, 0.5 represents a medium level, and 0.2 represents a low level. In combination with the parameter setting method in the literature by scholars such as Lin and Li (43) and Brand et al. (44), the intensity of local government regulation is also divided into three levels again: 0.8 represents a high level, 0.5 represents a medium level, and 0.2 represents a low level. On this basis, combined with other expert opinions, other relevant parameter values are proposed:


$$\begin{array}{l} \begin{array}{c} R_{ge}=12,C_{r}=5,S=3,R=3,T=2,P=8,R_{se}=1,L_{s}=1.2,\\ R_{f}=2,R_{j}=5,C_{P}=1,\\ C_{f}=1.5,C_{m}=1 \end{array} \end{array}$$

### The impact of tripartite willingness on the evolution of the system

**Figure 1:**
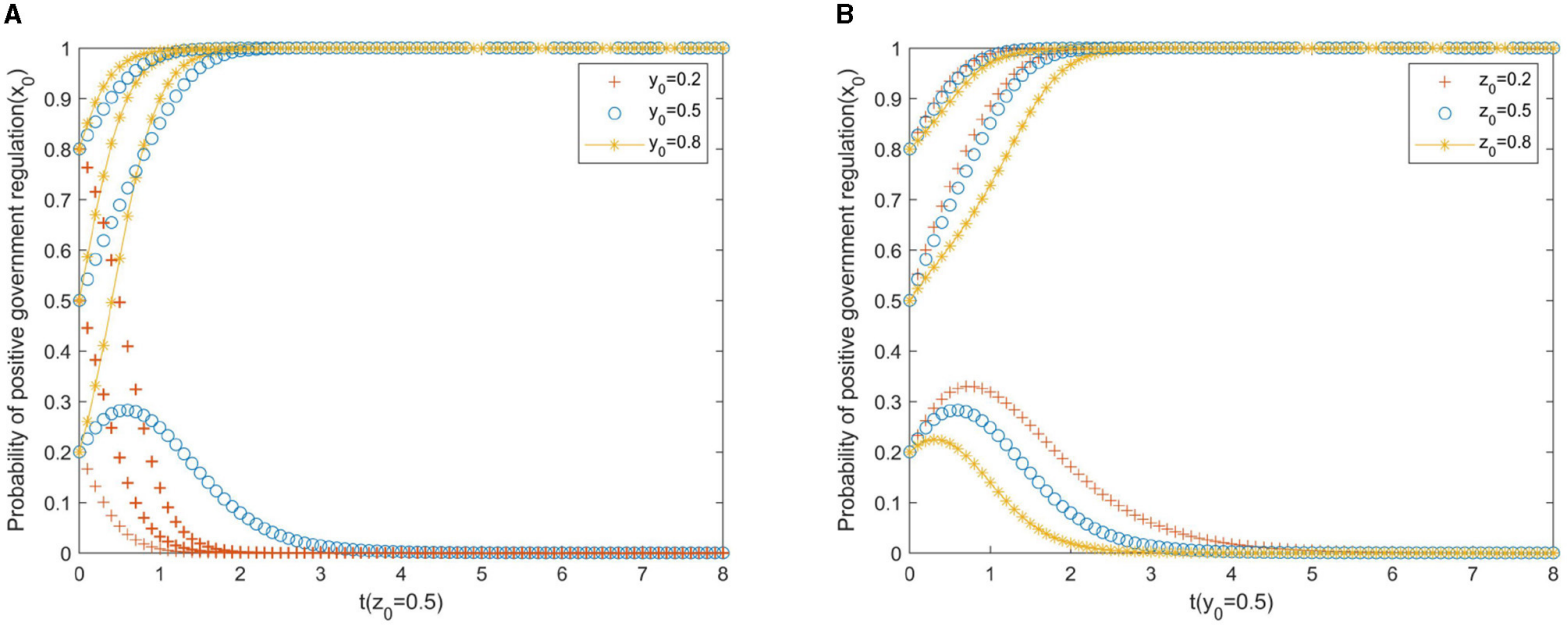
**(A)** Private sector. **(B)** Rural residents.

**Figure 2:**
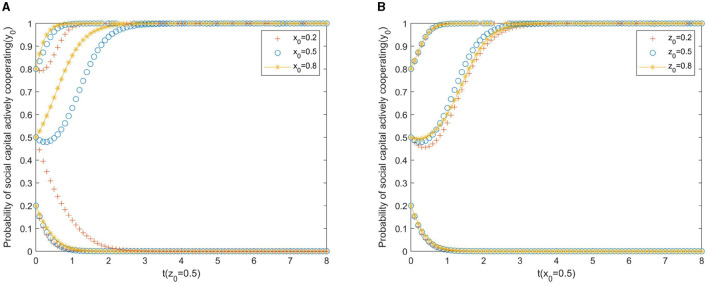
**(A)** Local government. **(B)** Rural residents.

**Figure 3:**
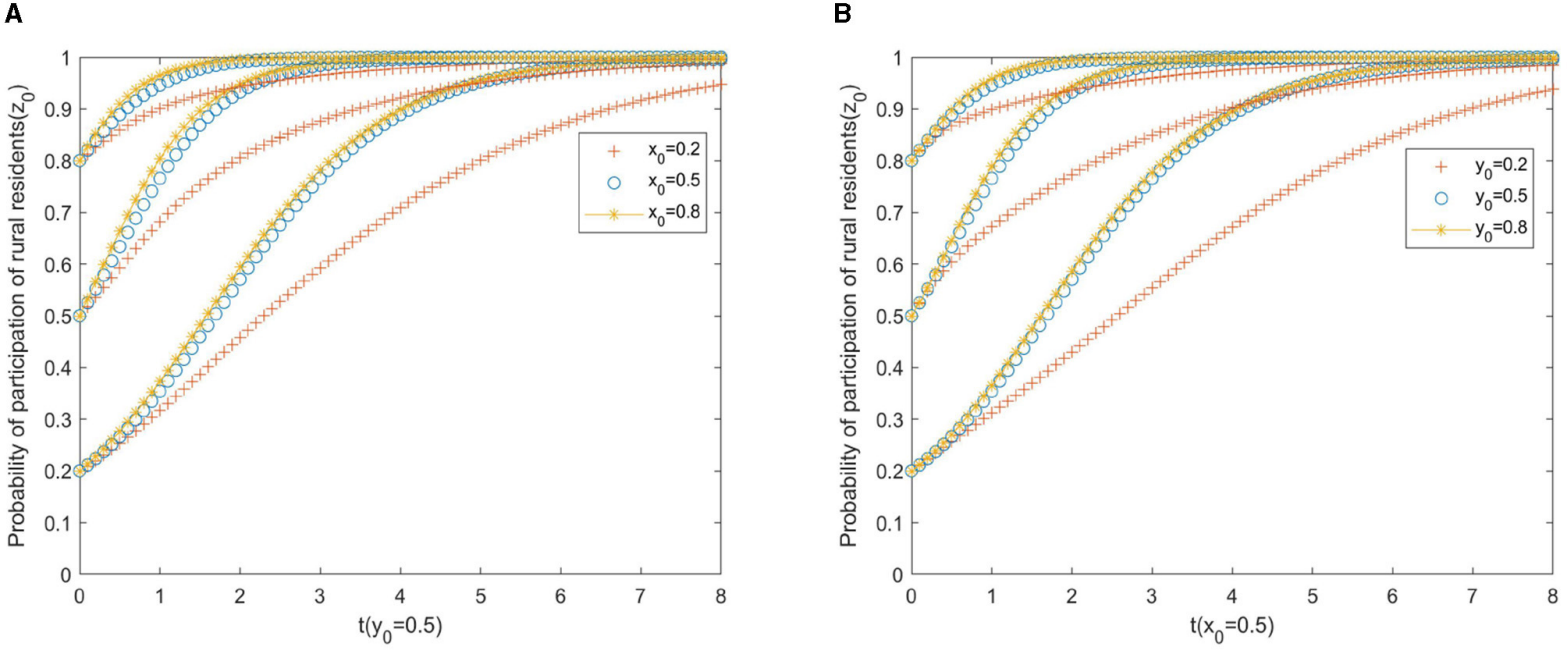
**(A)** Local government. **(B)** Private sector.

Based on the theoretical analysis under different situations, we now use the simulations performed on MATLAB 2017 to investigate the effects of punishment, subsidies to the private sector, and rural residents on the provision of older adult services. We assume that initial states of government, private sector, and residents belong to three levels, that is, *x*_0_, *y*_0_, *z*_0_∈(0.2, 0.5, 0.8). The parameters are initialized as displayed in Figures 1–**3**.

Figure 1 shows the impact of the participation of the private sector and rural residents on the strategic choices of local governments. As shown in Figure 1, the changes in the willingness of the private sector to invest capital and the willingness of rural residents to participate have an impact on the evolution of the behavioral strategies of local government, but not to the same extent. It can be seen from Figure 1A that where the initial willingness of local government to actively regulate is at a low or medium level (*x*_0_ ≤ 0.5), the government gradually evolves from negative to positive regulation with the increase in the initial willingness of the private sector to invest capital. In cases where the initial willingness of the private sector to invest capital is weak (*y*_0_ = 0.2), the government will always adopt negative regulation. In the same way, Figure 1B represents the changes in the willingness of rural residents to participate and the willingness of the government to regulate. If(*x*_0_ = 0.2), that is, the initial willingness of the government to promote positive regulation is low, no matter how the intensity of rural residents' participation changes, the local government will relax the policy incentives for rural residents, eventually evolving into a negative regulation. If the initial willingness of the government to take an active role in regulation is high (*x*_0_≥0.5), even if the initial willingness of rural residents to participate is weak, with policy encouragement, the evolutionary results will eventually move in the direction of active cooperation. Overall, the increase in the initial willingness of local government to encourage and regulate has a great impact on the promotion of rural PPP older adult care projects, and the increase in the initial willingness of the private sector and rural residents, who are the two main actors in the countryside, helps ensure coordinated cooperation in the project.

Figure 2 shows the impact of the participation of the local government and rural residents on the strategic choices of local governments. As Figure 2 shows, the improvement of active regulation by local government and the initial willingness of rural residents to participate influenced the evolution of the behavioral strategies of the private sector to invest capital. Compared to the initial willingness of rural residents to participate, the initial willingness of government regulatory efforts is more significant for the direction of evolution. Therefore, it is necessary to further promote the role of government regulation in guiding the private sector in the process of implementing rural PPP older adult care projects. Specifically, as shown in Figure 2A, if the initial willingness of the private sector to actively cooperate is weak (*y*_0_ = 0.2), whatever regulation strategy the local government adopts, the evolutionary result will move in the direction of negative cooperation. If the initial willingness of the private sector to actively cooperate is at a medium level (*y*_0_ = 0.5), increasing the initial willingness of local governments to regulate can enable evolution to move in the direction of positive cooperation. As can be seen from Figure 2B, if the initial willingness of the private sector to actively cooperate is low (*y*_0_ = 0.2), rural residents' willingness to participate has a small impact on the private sector's strategy, and the evolutionary result will move in the direction of negative cooperation. If the initial willingness of the private sector to actively cooperate is strong (*y*_0_≥0.5), even if the initial willingness of rural residents to participate is weak, the evolutionary results will evolve into active cooperation.

Figure 3 shows the impact of the participation of the local government and private sector on the strategic choices of local governments. As can be seen from Figure 3, the willingness of local government to regulate and the initial willingness of the private sector to invest capital affect the evolution of rural residents' behavioral strategies, and the effects of both are almost the same. As shown in Figures 3A, B, the increased willingness of the government to regulate and the increased capital investment by the private sector all accelerated the evolution of rural residents toward participation, which indicates that the government can promote rural residents' participation in the construction of the project by vigorously publicizing the rural PPP older adult care project. At the same time, the active cooperation of the private sector can also promote the participation of rural residents.

From Figures 1–3, it can be seen that in the game system of a rural public–private older adult care project, the initial willingness of the three players has a certain influence on the evolutionary results. To ensure the healthy development of the project, it is necessary to improve the willingness of the two main parties, the “private sector” and “rural residents” on the supply and demand sides, to ensure the participation of multiple subjects in the PPP project. The government should actively play a leading role in the early stage of project development, improve its willingness to regulate incentives in the early stage, and strengthen the supervision of the private sector in PPP projects.

### Simulation analysis under different parameter conditions

**Figure 4:**
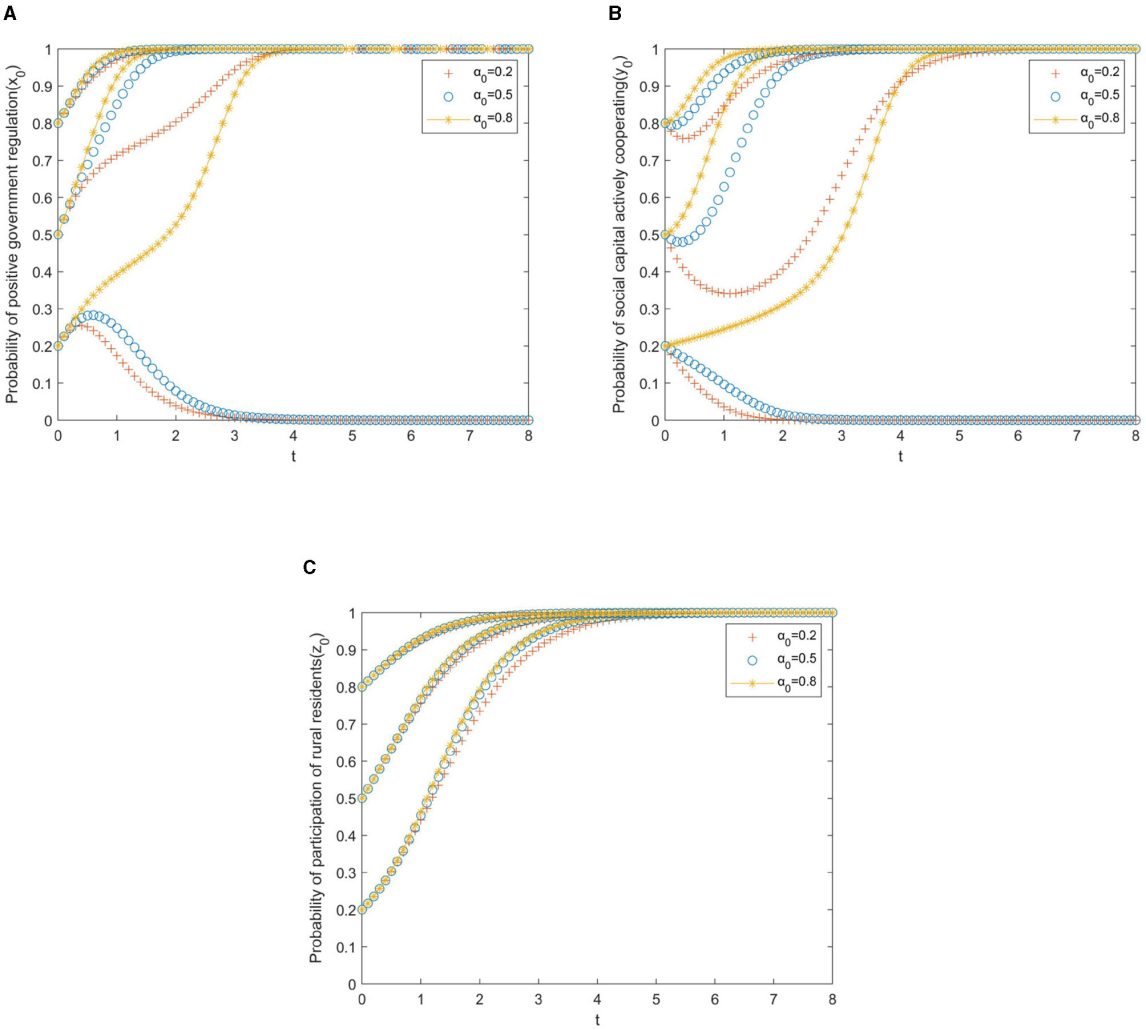
**(A)** Local government. **(B)** Private sector. **(C)** Rural residents.

**Figure 5:**
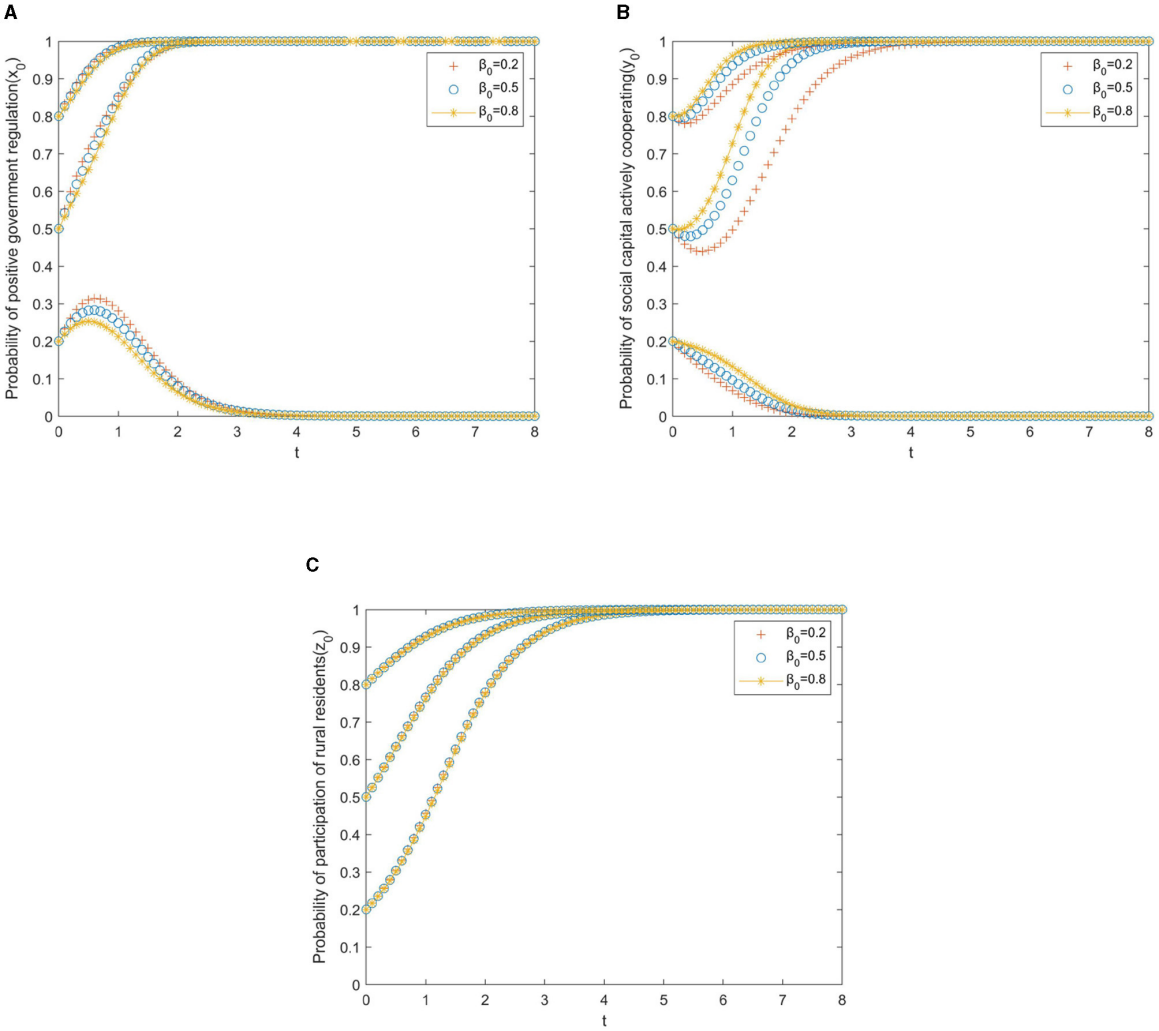
**(A)** Local government. **(B)** Private sector. **(C)** Rural residents.

**Figure 6:**
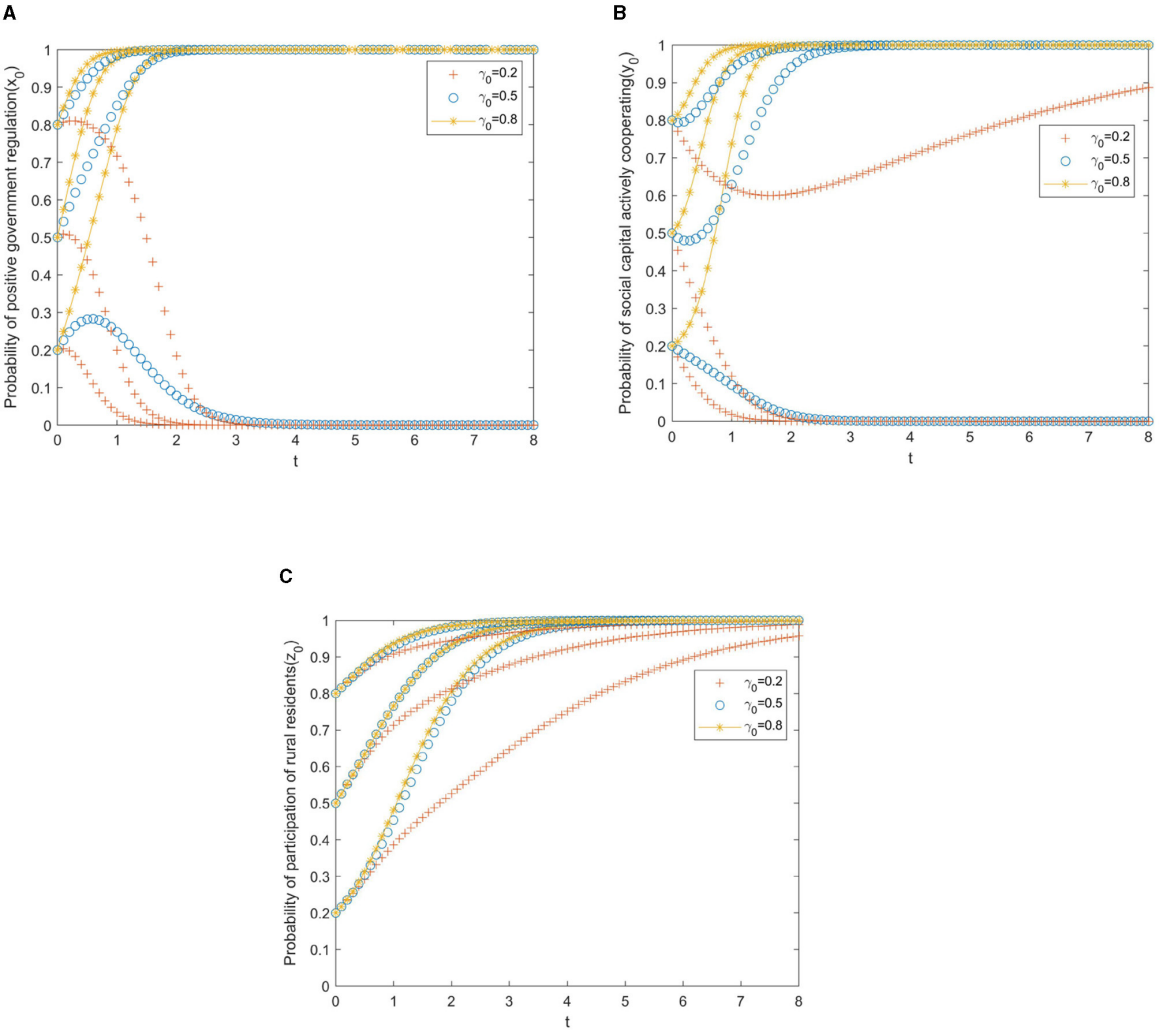
**(A)** Local government. **(B)** Private sector. **(C)** Rural residents.

**Figure 7:**
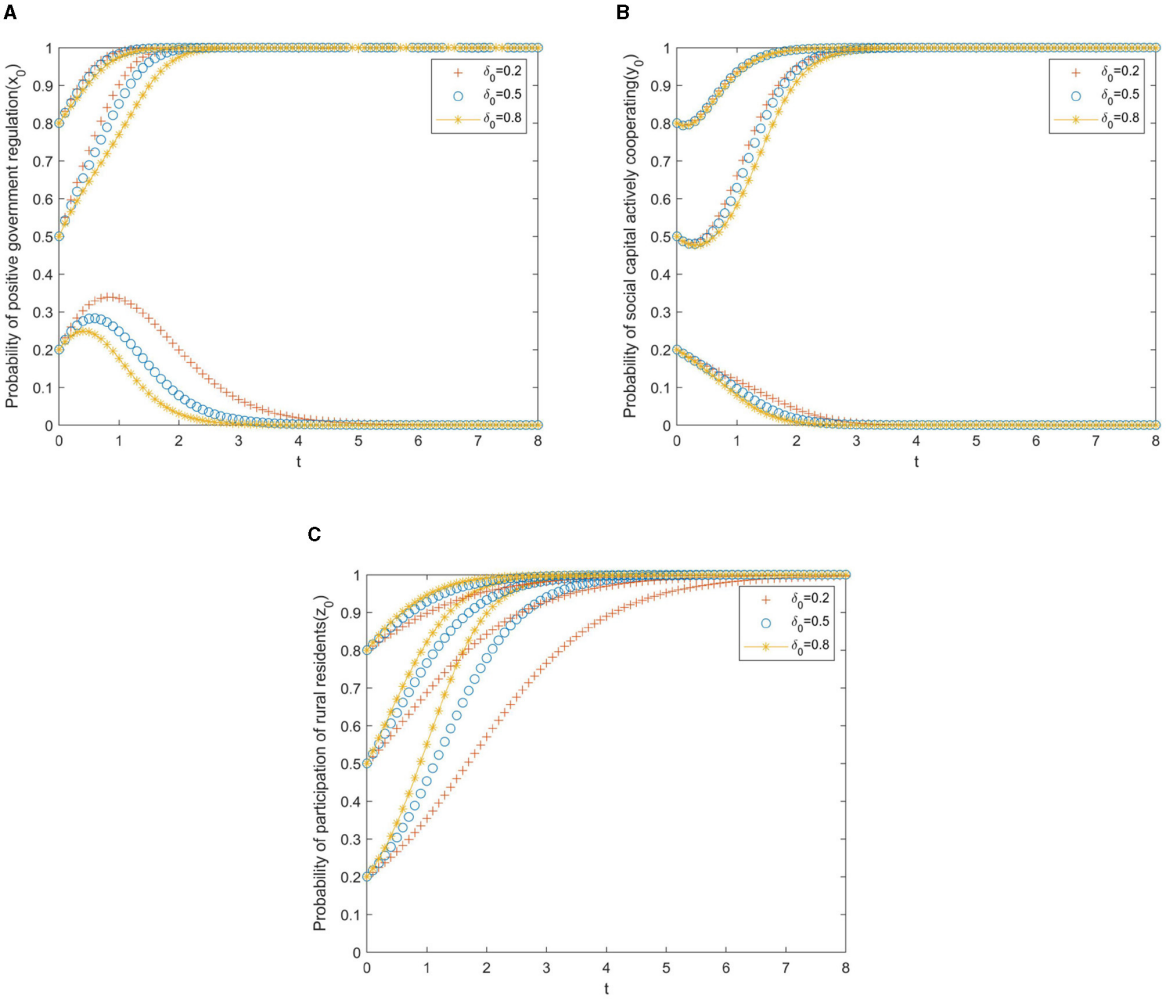
**(A)** Local government. **(B)** Private sector. **(C)** Rural residents.

In the simulation analysis, the simulation results will also be affected by the local government's reward and punishment measures for the private sector and the participation incentives for rural older adult residents. The strength of government rewards and punishments is divided into three levels. Thus, it can be obtained that: α, β, γ, δϵ(0.2, 0.5, 0.8), then the corresponding evolution trajectory is shown in Figures 4–7.

Figure 4 presents the evolutionary paths of the multi-subject system under different subsidies from the government to the private sector. In this case, the cooperative incentives β, the speculative punishments γ, and the participation incentives δ for rural residents are set to 0.5 to avoid the interference of other factors. As shown in Figure 4A, if the initial willingness of the government to promote positive regulation is weak (*x*_0_ = 0.2), the result of the game will evolve toward negative cooperation, and this evolution will slow down with the increase of government subsidies. It can be seen from Figure 4B that if the initial willingness of the private sector to actively cooperate is weak (*y*_0_ = 0.2), and the willingness of government subsidies to the private sector is at or below the medium level (α_0_ ≤ 0.5), the private sector will evolve in the direction of negative cooperation, but the evolution speed will slow down with the increase of government subsidies. From Figure 4C, we can observe that the increase in government subsidies has no significant impact on the evolution of rural residents' behavior strategies.

To sum up, the changes in government subsidies to the private sector have a weaker impact on the system's evolution than the changes in the initial willingness of the three parties. Therefore, in the process of improving older adult care, the government's subsidy policy to the private sector has reduced the cost of private sector investment to a certain extent, which ensures the motivation for cooperation and further accelerates the process of cooperation between the private sector and other players.

Figure 5 presents the evolutionary paths of the multi-subject system under different incentives from the government to the private sector. In the same way, the cooperative subsidies α, the speculative punishments γ, and the participation incentives δ for rural residents are set to 0.5 to avoid the interference of other factors. It is clear from Figure 5A that if the initial willingness of the government to adopt positive regulation is weak (*x*_0_ = 0.2), the PPP older adult care project will evolve toward negative collaboration. In addition, with the increase of local government incentives for the private sector, the evolution toward negative cooperation will accelerate. It can be drawn from Figure 5B that if the initial willingness of the private sector to actively cooperate is weak (*y*_0_ = 0.2), the private sector will evolve into negative cooperation. In this situation, the government's increasing incentives for private sector cooperation can slow down the speed of evolution. As can be seen from Figure 5C, different government incentives have no significant impact on the evolution of rural residents' behavioral strategies.

In short, compared with government subsidies, the influence of government incentives on institutional evolution is weaker. In this way, government incentives for private-sector cooperation can be used as a complementary means of subsidies to promote the adoption of positive cooperation strategies by the private sector.

Figure 6 presents the evolutionary paths of the multi-subject system under different punishments, from the government to the private sector. Likewise, the cooperative subsidies α, the cooperative incentives β, and the participation incentives δ for rural residents are set to 0.5 to avoid the interference of other factors. As shown in Figure 6A, if the willingness of the government to adopt a punishment strategy is low (γ = 0.2), no matter what the initial willingness of the government to positive regulation is, the PPP older adult care project will evolve toward negative collaboration. The reason may be that the punishment is weak and the benefits obtained by the government are less than the cost of regulation. We can learn from Figure 6B that if the initial willingness of the private sector to actively cooperate is weak (*y*_0_ ≤ 0.5), with the increases in government punishments, the private sector will shift from passive to active cooperation. As we can see from Figure 6C, the increase in government punishments has accelerated the evolution of rural residents' behavior strategies toward participation.

In general, in promoting the positive cooperation of the private sector, the government punishment strategy is better than the government reward strategy but lower than the government subsidy strategy, indicating that simple punishment measures can only promote the positive cooperation of the private sector to a certain extent. To better realize the active cooperation of the private sector, government subsidies and penalties should be combined to enhance the policy role of government regulation.

Figure 7 presents the evolutionary paths of the multi-subject system under different participation incentives from the government to rural residents. Similarly, the cooperative subsidies α, the cooperative incentives β, and the speculative punishments γ for the private sector are set to 0.5 to avoid the interference of other factors. It can be seen from Figure 7A that if the initial willingness of the government to adopt positive regulation is weak (*x*_0_ = 0.2), the PPP older adult care project will evolve toward negative collaboration. Moreover, with the increase in local government incentives for rural residents, the evolution toward negative cooperation will accelerate. As shown in Figure 7B, if the initial willingness of the private sector to take an active cooperation strategy is weak (*y*_0_ = 0.2), the government will increase the incentives for rural residents to participate, which will accelerate the evolution of the private sector to negative cooperation. The reason may be that after being awarded by the government, many rural residents chose to participate in the project, which increased the cost of active cooperation from the private sector. It is obvious from Figure 7C that the increase in government incentives for rural residents has promoted the evolution of rural residents' behavior strategies toward participation.

Overall, the government's incentive measures for rural residents have less influence on the system's evolution than the government's incentive measures for the private sector.

## Conclusion and policy suggestions

Based on the bounded rationality of participants, this study develops an evolutionary game model among local government, private sector, and rural residents to investigate the problem of the provision of quality services in the older adult care service industry using an agent-based computational approach. Specifically, this study describes the initial willingness of local government to actively regulate, the private sector to actively cooperate, and rural residents to participate in the three parties and constructs a three-party evolutionary game model. Then, through a simulation experiment, this study discusses the initial willingness of the tripartite subjects and the impact of government regulation on the evolution of the tripartite behavior strategy, and the conclusions are as follows:

1. In the PPP older adult care project, the four government regulation strategies of government subsidies to the private sector, speculative penalties, cooperative incentives, and government incentives to rural older adult groups have a decreasing impact on the evolution of the gaming system in order, and the impact is moderated by the initial willingness of the game subjects.
2. The initial willingness of the game players has a certain impact on the evolution of the system. Compared with the increase in local government subsidies and penalties for the private sector, the local government has improved the active supervision of the initial willingness and promoted the smooth implementation of the PPP older adult care project, especially in the early stages of implementation. Improving the local government's willingness to actively supervise the PPP older adult care project is an important guarantee for the smooth development of the project.
3. The local governments' incentives for rural residents have promoted rural residents to participate in the PPP older adult care project to a certain extent, but the initial willingness of rural residents to participate has a greater impact on their behavior strategies. Reducing the cost of rural residents' participation is the key to improving their initial willingness to participate.

The PPP older adult care project is still in the early stages of practice in the rural older adult care field. To promote the application and development of the project in the rural older adult care field and the sustainable improvement of rural older adult care security, the following suggestions are made:

- The government should use effective regulatory measures to improve government governance. The complexity of the PPP older adult care project and the conflicting interests of the participating subjects have put forward higher requirements for the governance capacity of local government, which should standardize the assessment mechanism, divide the responsibilities of the relevant interest subjects, formulate effective regulatory policies in combination with their affordable regulatory costs, and improve the deficiencies of the market mechanism in the aspect of regulation.
- The government should adopt a regulatory strategy that combines subsidy and penalty strategies. Both the single subsidy policy and the penalty policy are too one-sided to give full play to the role of government regulation in the PPP older adult care project. Local government subsidies increase the motivation of the private sector to a certain extent, but they also reduce the market competitiveness of service providers in the rural older adult care field and may induce speculative behavior, which should be regulated by a combination of penalty strategies. Local governments should formulate flexible subsidy policies and dynamic penalty policies to promote the participation of the private sector in rural older adult care services and restrain their speculative behavior.
- The government should increase the benefits of rural residents' participation and take the main role of rural residents into account. Rural residents are the beneficiaries of the project and are also important participants in the PPP older adult care project. Local government should increase the publicity of the rural PPP older adult care project, develop incentives for rural residents to participate, increase the initial willingness of rural residents to participate, allow rural residents to actively participate in the project, and take on the responsibility of supervision to ensure that the project are more in line with the requirements of the residents.

This study considers the impact of factors such as the subject's willingness to participate and the local government's reward and punishment mechanism on the development of rural PPP older adult care projects, which is of some reference significance to the management of rural PPP older adult care projects and the formulation of policies. However, at present, the development of rural PPP older adult care projects is at an early stage, and data collection is a difficult problem. On the contrary, this study is a simple division of rural residents' participation into participation and non-participation, and there may be cases of participation and withdrawal in the actual situation, which may lead to different conclusions from a dynamic perspective.

## Data availability statement

The original contributions presented in the study are included in the article/supplementary material, further inquiries can be directed to the corresponding author.

## Author contributions

JF and CH: conceptualization. JF: methodology, investigation, writing and editing, oversight, and funding acquisition. CH: formal analysis and data management, paper writer, and idea provider. SL: resources and visualization. YX: project management. All authors contributed to the article and approved the submitted version.
